# The contribution of peroxynitrite generation in HIV replication in human primary macrophages

**DOI:** 10.1186/1742-4690-4-76

**Published:** 2007-10-21

**Authors:** Stefano Aquaro, Carolina Muscoli, Alessandro Ranazzi, Michela Pollicita, Teresa Granato, Laura Masuelli, Andrea Modesti, Carlo-Federico Perno, Vincenzo Mollace

**Affiliations:** 1 Department of Experimental Medicine and Biochemical Sciences, University of Tor Vergata, Rome, Italy; 2 Department of Pharmaco-Biology, University of Calabria, Rende(CS), Italy; 3 Faculty of Pharmacy, University of Catanzaro "Magna Graecia", Roccelletta di Borgia, Catanzaro, Italy; 4 San Raffaele Pisana IRCCS, Rome, Italy; 5 IBPM-CNR, Rome, Italy; 6 Istitute Mondino-Tor Vergata, Rome, Italy

## Abstract

**Background:**

Monocytes/Macrophages (M/M) play a pivotal role as a source of virus during the whole course of HIV-1 infection. Enhanced oxidative stress is involved in the pathogenesis of HIV-1 infection. HIV-1 regulatory proteins induce a reduction of the expression and the activity of MnSOD, the mitochondrial isoform leading to a sustained generation of superoxide anions and peroxynitrite that represent important mediators of HIV-1 replication in M/M. MnTBAP (Mn(III)tetrakis(4-benzoic acid)porphrin chloride), a synthetic peroxynitrite decomposition catalyst, reduced oxidative stress subsequent to peroxynitrite generation.

**Results:**

Virus production was assessed by p24 ELISA, western blot, and electron microscopy during treatment with MnTBAP. MnTBAP treatment showed a reduction of HIV-1 replication in both acutely and chronically infected M/M: 99% and 90% inhibition of p24 released in supernatants compared to controls, respectively. Maturation of p55 and p24 was strongly inhibited by MnTBAP in both acutely and chronically infected M/M. EC_50 _and EC_90 _are 3.7 (± 0.05) μM and 19.5 (± 0.5) μM, in acutely infected M/M; 6.3 (± 0.003) μM and 30 (± 0.6) μM, in chronically infected M/M. In acutely infected peripheral blood limphocytes (PBL), EC_50 _and EC_90 _are 7.4 (± 0.06) μM and of 21.3 (± 0.6) μM, respectively. Treatment of acutely-infected M/M with MnTBAP inhibited the elevated levels of malonildialdehyde (MDA) together with the nitrotyrosine staining observed during HIV-1 replication. MnTBAP strongly reduced HIV-1 particles in infected M/M, as shown by electron microscopy. Moreover, in presence of MnTBAP, HIV-1 infectivity was reduced of about 1 log compared to control.

**Conclusion:**

Results support the role of superoxide anions in HIV-1 replication in M/M and suggest that MnTBAP may counteract HIV-1 replication in combination with other antiretroviral treatments.

## Background

It is well know that monocytes/macrophages (M/M) are important targets of human immunodeficiency virus type 1 (HIV-1) in infected patients. Such cells are widely recognized to play a pivotal role as a source of virus during the whole course of HIV-1 infection, even in patients receiving antiretroviral therapy [1]. HIV-1 infection does not lead to M/M depletion as occurs for HIV-1 infected CD4^+ ^T-lymphocytes; instead, once infected by HIV-1, M/M produce large amounts of infectious viral particles for a long period of time [2]. Productively infected M/M can fuse with CD4^+ ^T-lymphocytes and transfer the virus to these cells within the context of antigen presentation [3]; in addition, infected M/M are able to trigger apoptosis of T-lymphocytes (either CD4^+ ^or CD8^+^) [4-6] as well as astrocytes, [7-9]. Few HIV-infected M/M are sufficient to induce the recruitment and activation of HIV-infected resting CD4^+ ^lymphocytes [10] and infect resting CD4^+ ^T-lymphocytes [11]. Recently, it has been demonstrated that as few as 500 HIV-exposed M/M cause complete depletion of several millions of autologous CD4+ T-lymphocytes, sustained HIV-viremia and spreading of HIV-1-DNA in mouse lymphoid organs [12]. Therefore, M/M sustain persistent and continuously productive HIV infection [13]. Moreover, evidence exists suggesting that enhanced oxidative stress may be involved in the pathogenesis of HIV infection and HIV-1-infected patients are under chronic oxidative stress [14-16]. The activation of CD4^+ ^T-lymphocytes and M/M, which occurs during HIV infection, is most likely due to increased production of free radicals such as superoxide anion, peroxynitrite (the by-product of super oxide and nitric oxide, NO) and hydroxyl radical which is generated by peroxynitrite decomposition [8,17]. In addition, elevated serum levels of hydroperoxides and malondialdehyde (MDA), which are recognized as markers of lipid peroxidation subsequent to free radical overproduction, have also been found in asymptomatic HIV-1-infected patients early in the course of the disease [18]. Under normal circumstances, in healthy individuals, the free radicals burden is highly regulated by the endogenous antioxidant systems (i.e. superoxide dismutase enzymes, SOD) and glutathione peroxidase. Previous studies suggest that oxidative stress plays a crucial role during HIV-1 pathogenesis, including viral replication, inflammatory response, decreased immune cell proliferation, loss of immune function, chronic weight loss and increased sensitivity to drug toxicity. In particular, the disruption of oxidative status contributes to the cell damage observed during HIV-1 infection, yet it is worth stressing that such alteration is particularly relevant in M/M [18,19]. The alteration of the homeostasis induced by HIV-1 infection in M/M, with consequent production of toxic factors, is claimed to be the main cause of neuronal damage during AIDS [17]. In particular, the release of some coating component of HIV-1, such as gp120 glycoprotein by HIV-1-infected M/M produces both direct and indirect effects in the central nervous system (CNS) and in the systemic compartment [20,21]. The neuronal injury can result from a direct mechanism by interaction with viral proteins, such as gp120, Tat (Transcriptional transactivator) and Vpr (viral protein R) produced by infected cells, or by an indirect effect resulting from the inflammatory process involving activated, though not necessarily HIV-1-infected, monocytes, macrophages and astrocytes [22]. Furthermore, HIV-1 infection induces a significant perturbation of oxidative status of M/M associated with an increased production of MDA and a decreased synthesis of endogenous glutathione [18]. In addition, the HIV regulatory proteins induce a reduction of expression and activity of MnSOD, the mitochondrial isoform of the enzyme [23], leading to a sustained generation of superoxide and peroxynitrite and in turn imbalance of the cellular homeostasis [24,25].

The associations of HIV infection with the formation of free radical species led us to hypothesize that superoxide and peroxynitrite may represent important mediators of M/M-HIV replication. To test this, we employed MnTBAP (Mn(III)tetrakis(4-benzoic acid)porphrin chloride), a synthetic peroxynitrite decomposition catalyst proven to reduce oxidative stress subsequent to peroxynitrite generation [9,26].

## Results

### Acutely-infected macrophages

**Figure 1:**
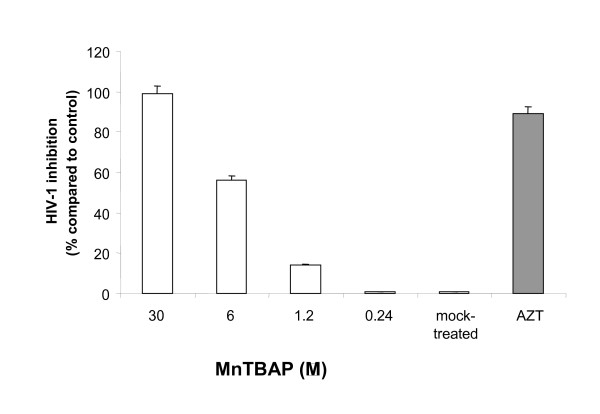
**Antiviral activity of MnTBAP in acutely HIV-1 infected macrophages**. Monocytes were cultured for 5 days to generate monocyte-derived macrophages which where infected with 300 TCID50/ml HIV-1 BaL and treated acutely (i.e. treated with drugs prior to virus challenge). Supernatants were collected day 14 after infection and tested for virus production by analysis of HIV-1 p24 gag Ag production with a commercially available kit ELISA.

A significant dose dependent antiviral activity of MnTBAP was achieved in acutely-infected M/M (i.e. treated with drugs prior to virus challenge). In particular, the 1.2 μM dose of MnTBAP led to a reduction of p24 gag Ag production down to about 15% (Fig. 1). Indeed, concentration of 30 μM completely inhibited HIV-1 replication up to day 14, end of experiment, affording undetectable levels of p24 gag Ag production (Fig. 1), no signs of toxicity were observed at 30 μM (data not shown). Interestingly, virus inhibition remained constant for all concentrations tested up to day 14 (data not shown). Therefore, neither major breakthrough nor cumulative inhibition of virus replication occurred in acutely-infected M/M at least up to 14 days after infection. EC_50 _and EC_90 _were then calculated and found to be 3.7 (± 0.05) μM and 19.5 (± 0.5) μM, respectively (Table 1).

**Table 1:** Comparative anti-HIV-1 efficacy of MnTBAP in macrophages and lymphocytes

Cells	EC_50 _(mM)	EC_90 _(mM)	TC_50 _(mM)
**Macrophages**			
Acutely infected	3.7 (± 0.05)	19.5 (± 0.5)	60
Cronically infected	6.3 (± 0.003)	30 (± 0.6)	60
**Limphocytes**			
Acutely infected	7.4 (± 0.06)	21.3 (± 0.6)	50

The geometric mean of p24 gag Ag production of replicates in each experiment was used to determine the effective drug concentration where 50% and 90% of viral replication is inhibited (EC50 and EC90, respectively), by linear regression of the log of the percent HIV-1-p24 production (compared to untreated controls) versus the log of the drug concentration. TC50: toxic concentration 50%; drug toxicity has been assessed in the absence of viral infection.

### Chronically-infected macrophages

**Figure 2:**
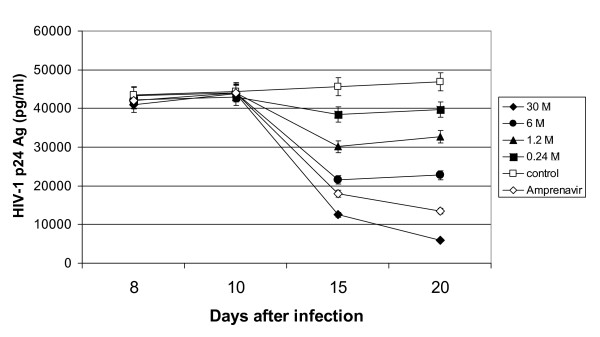
**Antiviral activity of MnTBAP in chronically HIV-1 infected macrophages**. Monocytes were cultured for 5 days to generate monocyte-derived macrophages which where infected with 300 TCID50/ml HIV-1 BaL and treated chronically (i.e. treated with drugs 10 days after infection) with MnTBAP at indicated doses, and Amprenavir (4 uM). Supernatants were collected at day 8, 10, 15, 20 after infection and tested for virus production by analysis of HIV-1 p24 gag Ag production with a commercially available kit ELISA.

To study the activity of MnTBAP in chronically-infected M/M, antiviral treatment was started 10 days after infection, when both HIV-1 p24 gag Ag (Fig. 2) and genomic HIV-RNA (data not shown) released in the supernatants show a stable virus production. A decrease in the release of mature proteins, compared to control, was already detectable by day 5 after drug treatment with the highest concentrations of MnTBAP (30 μM and 6 μM) (Fig. 2), and become more pronounced at day 10 (30 μM). This event was similar to the inhibition observed when amprenavir (4 μM), a protease inhibitor employed as control of chronic inhibition, was employed. Starting from day 5 of drug treatment, and until the end of the experiment, a quasi-stable and substantial inhibition of the release of HIV-1 p24 gag Ag was detected with concentrations of MnTBAP of 6 and 30 μM (about 50% and 90% at day 5, respectively) up to day 10 after treatment. No complete inhibition of virus replication could be achieved at the highest non-toxic concentrations tested (Fig. 2). Based on these data, EC_50 _and EC_90 _of MnTBAP were 6.3 (± 0.003) μM and 30 (± 0.6) μM, respectively (Table 1). Treatment with 10 μM of AZT (about 200-fold greater than its EC_90 _in HIV-1-acutely infected M/M) was not able to reduce the production of HIV-1-p24 gag Ag in chronically infected M/M (data not shown). This further confirms the absence of new rounds of replication in these cells after day 10 of infection [2,27].

### Acutely-infected PBL

We wished to compare these results with those obtained using protease inhibitors in PBL. MnTBAP has shown a stable antiviral activity in acutely infected PBL until the end of the experiment (day 10 after infection), with an EC_50 _of 7.4 (± 0.06) μM and an EC_90 _of 21.3 (± 0.6) μM (Table 1). These EC_50 _and EC_90 _are and in the range (or lower in the case of EC_90_) of those determined in acutely infected M/M (Table 1).

### Drug toxicity

Treatment of M/M and PBL with concentrations of MnTBAP showed no decrease in cell number, thus suggesting the absolute absence of major toxicity at used concentrations (Table 1). Thus, the antiviral activity observed in these experiments can be attributed only to the MnTBAP inhibitory effect.

### HIV-1 p24 and p55 gag proteins analysis

**Figure 3:**
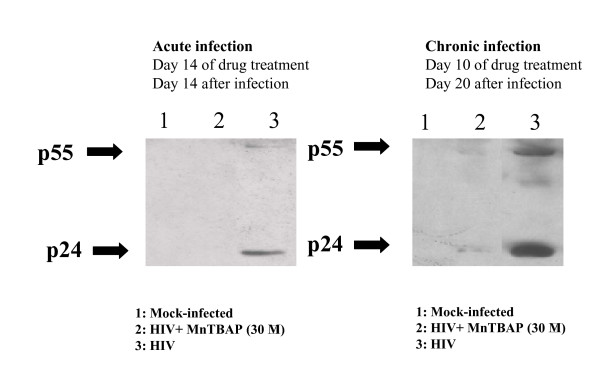
**MnTBAP reduces p24 and p55 expression in HIV-infected macrophages**. Western blots of lysates of acutely and chronically infected M/M. Line 1: Mock-infected macrophages. Line 2: macrophages HIV-1 BaL infected and treated with MnTBAP (30 μM). Line 3: macrophages HIV-1 BaL infected.

The western blots of lysates of acutely and chronically infected M/M treated with MnTBAP are shown in Fig. 3. When the M/M lysates were examined, the inhibition of HIV-1-p24 antigen release into the supernatants correlated with the disappearance of the p24 band in the immunoblots in both acutely and chronically infected M/M. Interestingly, HIV-1 p55 antigen was also inhibited by MnTBAP treatment (Fig. 3) showing that MnTBAP is able to counteract both the p24 as the precursor p55 formation, revealing the ability of MnTBAP to block the maturation of p24 viral protein.

### Selective inactivation of peroxynitrite in HIV-1 infected macrophges

**Figure 4:**
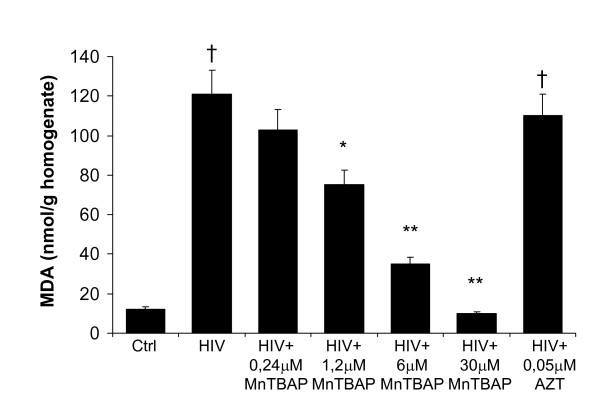
**MnTBAP inhibits MDA in HIV-infected macrophages in a dose dependent fashion**. MDA increased within HIV-1-infected macrophages. Treatment with MnTBAP (0,24–30 μM) antagonized MDA overproduction dose-dependently while AZT (0.05 μM) was not able to inhibit macrophages HIV-related MDA formation. † P < 0.001 when compared to control; * P < 0.05 and ** P < 0.001 when compared to HIV-infected cells.

**Figure 5:**
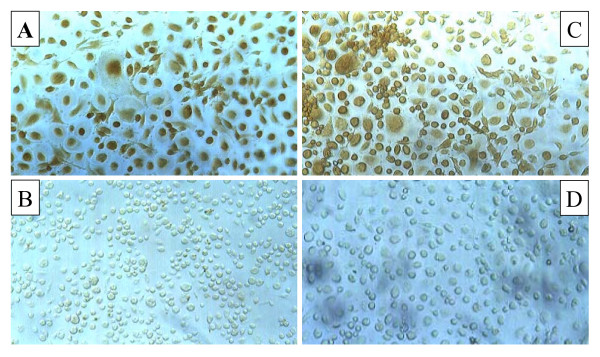
**MnTBAP inhibits nitrotyrosine formation in HIV-infected macrophages**. Photomicrographs (optical microscopy) of nitrotyrosine staining in HIV-1-infected macrophages. HIV-1 infection enhance the immunocytochemical expression of nitrotyrosine (Panel A) in compared to mock-infected macrophages (Panel B), indicating an increased production of peroxynitrite. Acute treatment with MnTBAP (30 μM) (Panel D), but not with AZT (0.05 μM) (Panel C) is able to inhibit in macrophages HIV-related peroxynitrite formation.

Furthermore, HIV-1 replication was associated with an increase of MDA level and nitrotyrosine staining indicating the HIV-related peroxynitrite formation (Fig. 4, 5A) as evaluated 14 days after HIV-infection. Treatment of acutely-infected M/M with MnTBAP (0,24–30 μM) inhibited the MDA formation in a dose response manner (Fig. 4) and nitrotyrosine staining observed during HIV-1 replication (30 μM; Fig. 5D) by removing the superoxide and peroxynitrite formation. As a control and consistent with previously published data, 0.05 μM AZT induced about 90% inhibition of virus replication in these acutely infected M/M (Fig. 1), but did not affected the nitrotyrosine staining nor MDA level (Fig. 4, 5C).

### Effects of MnTBAP upon virus infectivity

**Figure 6:**
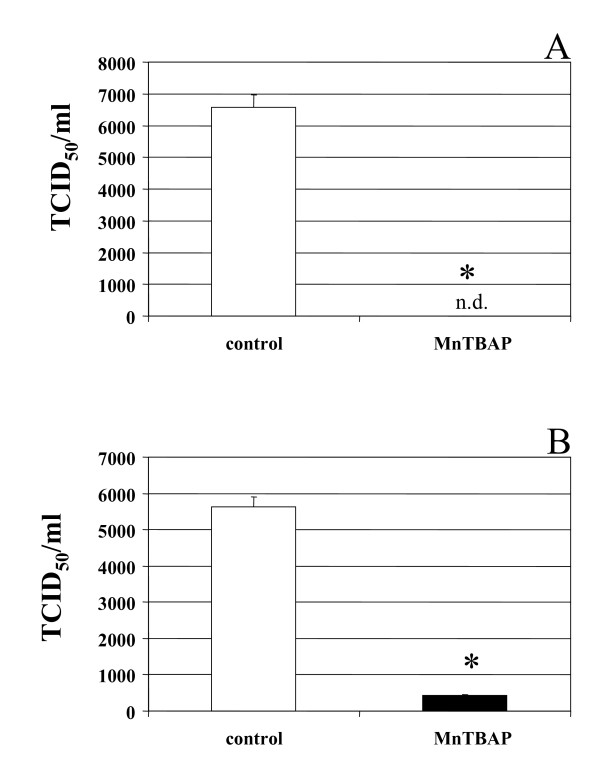
**Removal of free radicals by MnTBAP is involved in macrophages HIV replication**. Infectivity of virus particles produced by HIV-1-infected macrophages was evaluated on macrophages obtained from a different seronegative donor exposed to serial dilution of supernatants from MnTBAP treated or not-treated HIV-1-infected macrophages. The TCID50/ml was calculated according to Reed and Muench method. MnTBAP reduces TCID50 about a log both in acutelly (Panel A) as in chronically (Panel B) HIV-1-infected macrophages compared to infected and non treated macrophages.

We investigated the production of infectious virus particles by both acutely and chronically infected M/M. Supernatants of HIV-1 acutely and chronically infected M/M previously treated with MnTBAP 6 μM and 30 μM, respectively, were titered in cultures of M/M and compared with the infectivity of not treated HIV-1 infected M/M supernatants taken at the same time-point. The infectivity of supernatants of not treated HIV-1 acutely infected M/M had a titer of 6.57 × 10^3 ^TCID_50_/ml (Fig. 6A), while supernatants from MnTBAP (6 μM) treated HIV-infected M/M showed a not detectable infectivity (Fig. 6A). Similarly, the infectivity of supernatants of not treated HIV-1 chronically infected M/M had a titer of 5.62 × 10^3 ^TCID_50_/ml (Fig. 6B), while supernatants from MnTBAP (30 μM) treated HIV-infected M/M had a titer of 4.21 × 10^2 ^TCID_50_/ml (Fig. 6B), that means a reduction of more than 92% of infectivity.

### Ultrastructural analysis of acutelly-infected M/M treated with MnTBAP

**Figure 7:**
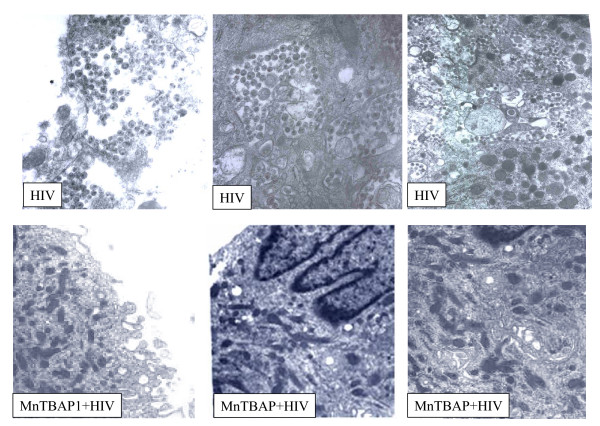
**Electron microscopy of untreated or MnTBAP-treated HIV-1 infected macrophages**. Untreated macrophages show accumulation of many mature particles, at different stages of maturation, in cytoplasmatic vacuoles and in the extracellular space. By contrast in MnTBAP (30 μM) treated macrophages no viral particles are found. This observation support the hypothesis that MnTBAP treatment is able to prevent enveloped and unenveloped virions production.

In order to better understand the mechanism through which the removal of peroxynitrite was acting on HIV-1 acutely infected M/M, electron microscopy was performed at day 14 after treatment. It is know that the mature HIV-1 particles are accumulated in intracytoplasmic vacuoles in M/M before the budding. As shown in Figure 7, MnTBAP at concentration of 6 μM dramatically reduced the presence of HIV-1 particles inside cytoplasmic vacuoles and in the extracellular compartment (Fig. 7). Moreover, the number of these cytoplasmic vacuoles was dimished by MnTBAP treatment in HIV-1-infected M/M, compared to untreated HIV-1-infected M/M. These observations might contribute to the profound infectivity reduction induced by MnTBAP treatment that is able to disturb the HIV-1 protein maturation.

## Discussion

The design of this study was based on the crucial importance of infected macrophages in the pathogenesis and progression of HIV-1 infection. During HIV-1 infection the imbalance of the intracellular redox status due to inflammatory stress has been previously reported [28]. Indeed, free radicals are generated following HIV infection of macrophages and microglia [1,12,17]. In particular, HIV-1 replication is enhanced under oxidative condition *in vitro *[29]. For example, *in vitro *HIV-1 infection of macrophages resulted in superoxide and peroxynitrite production [30,31]. Indeed, our findings have shown that immunohistochemical staining for nitrotyrosine, the footprint of peroxynitrite, showed extensive immunoreactivity in HIV-1 macrophages cytoplasm and this overproduction was counteracted by MnTBAP, but not by AZT treatment. In addition, HIV-infected macrophages presented elevated levels of malonildialdehyde, the biochemical marker of lipid peroxidation that were inhibited by MnTBAP treatment in a dose-dependent fashion.

Furthermore, significant and sustained inhibition of HIV-1 replication was obtained in both acutely and chronically infected macrophages by removing the overt production of peroxynitrite. The effect of MnTBAP, a peroxynitrite decomposition catalyst, upon both the core protein p24 and its precursor p55 suggests that that free radicals may interfere with HIV-1 protein expression in both acute and chronic infection thus suggesting the important role played by the oxidative status in HIV-1 replication. This is in agreement with previous observations which point out the role of oxidative stress in HIV-1 proteins maturation and folding in both lymphocytes and macrophages [32-34]. Moreover, western blot analysis revealed that MnTBAP, by inhibiting both the p24 as the p55 formation, is able to counteract not only the formation, but also the maturation of this core viral protein. To confirm that MnTBAP is able to act on the virus maturation the formation of mature viral particles in intracytoplasmic vacuoles of M/M has been analyzed by electron microscopy. It is known that the mature HIV-1 particles are accumulated in intracytoplasmic vacuoles in M/M before the budding. Indeed, electron microscopic analysis of intracellular compartments in HIV-1 infected M/M has shown that the number of mature virus particles was dramatically decreased by MnTBAP. Moreover, virus particles budding was also fully inhibited in agreement with previous studies where restoration of the oxidative status homeostasis led to the inhibition of HIV-1 budding in macrophage cells. These results so can indicate that MnTBAP disturbs the HIV-1 proteins maturation.

Lack of HIV-1 maturation is correlated to a dramatic reduction of virus infectivity. The production of infectious virus particles by both acutely and chronically infected M/M was strongly counteracted (a reduction of about 4 and 2 log, respectively) by MnTBAP treatment. Nevertheless, complete inhibition of HIV replication was not achieved. This is not surprising since all the HIV inhibitors are less (or even not) active in chronically-infected when compared with acutely-infected macrophages [27,35,36].

It can be hypothetized that the dramatic virus inhibition obtained by the employment of peroxynitrite decomposition catalyst is due to, at least in part, an indirect mechanism on NF-kB pathway. Indeed, it is well known that reactive oxygen species activate NF-kB that, in turn, is an obligatory step for HIV-1, together with several viruses, replication [[37,38], although further experiments are needed to confirm this hypothesis].

## Conclusion

However, other than this hypothesis, overall data presented in this article suggest that the inhibition of virus maturation and release can be related to a block of post-transcriptional/post-translational events of the virus life cycle. In fact, MnTBAP treatment substantially modify the expression of virus proteins in chronically (better in acutely) infected macrophages. This structural proteins are crucial for the infectivity of HIV-1. Nevertheless, we cannot exclude that other factors related to peroxynitrite inactivation influence the virus replication. In conclusion our results highlight the role of peroxynitrite generation in HIV replication in human macrophages and show that the removal of peroxynitrite by selective antioxidants such as peroxynitrite decomposition catalysts contribuits to the inhibition of HIV replication in macrophages, the cells acting as a reservoir of the virus. Furthermore, data here reported suggest the potential usefulness of these compounds alone or in association with other antiretrovirals and may represent the basis for alternative and efficient strategies for the treatment of HIV-1 infection.

## Methods

### Compounds

The peroxynitrite decomposition catalyst MnTBAP was purchased from Alexisis Biochemicals (Switzerland). 3'-azido-2', 3'-dideoxythymidine (AZT), an inhibitor of HIV replication, was used as control at concentrations known to be active against HIV-1 replication. All compounds and reagents (with the exception of MnTBAP) were obtained from Sigma (St. Louis, USA). The nucleoside analogue reverse transcriptase inhibitor AZT was diluted in PBS and stored at -80°C before using.

### Cell cultures

#### Macrophages and lymphocytes

Primary M/M were prepared and purified as previously described [39]. Briefly, PBMC obtained from healthy HIV-1-negative donors were separated over Ficoll gradient and seeded in 48-well plates or in glass chamber (for immunocytochemical analysis) at 1.8 × 10^6 ^cells/well in 1 ml of RPMI 1640 containing 20% heat-inactivated, endotoxin and mycoplasma-free fetal bovine serum (Hyclone Laboratories, Inc., Logan, UT), 4 mM L-glutamine (Life Technologies), 50 U/ml penicillin and 50 μg/ml streptomycin (Life Technologies) (herein after referred to as complete medium). Five days after plating and culturing the PBMC at 37°C in a humidified atmosphere enriched with 5% CO_2_, non-adherent cells were carefully removed by repeated washings with warmed RPMI 1640, leaving a monolayer of adherent cells which were finally incubated in complete medium. Cells treated under these conditions have been shown to be >97% M/M, as determined by cytofluorimetric analysis [39,40].

Peripheral blood lymphocytes (PBL) were purified from PBMC by repeated adherences to remove monocytes, and then cultured with the same medium as M/M, supplemented with 2 μg/ml phytohemagglutinin (PHA). Stimulation was carried out for 72 hours; afterward, the medium was discarded, cells were washed three times with RPMI 1640 and the concentration was adjusted to 5 × 10^5 ^cells per ml of medium supplemented with 50 U/ml recombinant interleukin-2 (IL-2).

#### HIV-1 isolates

Two different viral isolates of HIV-1 were used in this study. A monocytotropic isolate of HIV-1 such as HIV-1_BaL _was used in all experiments involving primary M/M. Characteristics and genomic sequence of this strain have been previously described [41-44]. The virus was expanded in M/M, whose supernatants were collected, filtered and stored at -80°C before use [44]. Characteristics of viral stocks used for this study were 2.1 × 10^8 ^HIV-RNA genomes/ml (corresponding to 35 ng of p24 antigen) and 5 × 10^3 ^tissue culture infectious doses 50% per ml (TCID_50_/ml) as assessed by virus titration in other primary M/M cultures. The prototypic lymphocytotropic strain of HIV-1, named HIV-1_IIIB _and used to infect PBL, was obtained from acutely infected H9 CD4^+ ^T-lymphocytes cell line, and then expanded in PBMC. Cell free virus present in the supernatants was collected, ultracentrifuged, filtered (0,22 μM) and stored at -80°C. Titre of HIV-1_IIIB _viral stocks used in this study was 5 × 10^6 ^TCID_50_/ml, as assessed in CD4^+ ^T-lymphocytic cell line C8166.

#### Drug toxicity

M/M and PBL were treated for 14 to 21 days in the presence of different concentrations of MnTBAP. Cell viability of M/M and PBL was visually assessed (and compared to untreated controls) using the trypan blue exclusion method. Briefly, cells were exposed to dye, and then visually examined to determine whether cells take up or exclude dye. The live cells that possess intact cell membranes exclude trypan blue, whereas dead cells do not. Drug toxicity was assessed in the absence of viral infection.

#### Assessment of drug activity in acutely infected M/M

One day after separation (i.e. 6 days after plating), M/M were treated with various concentrations of drugs (MnTBAP, 0.24, 1.2, 6 and 30 μM; AZT, 0.05 μM), and then exposed to 300 TCID_50_/ml of HIV-1_Ba-L _(a virus dose affording a maximal virus production from M/M). Two hours after virus challenge, M/M were washed to remove the viral inoculum, and complete medium containing the appropriate drugs was replaced. Macrophages were then cultured for the duration of the experiments by refunding them with fresh complete medium and drugs every 2 days. Supernatants were collected at different time points for assessment of virus production by analysis of HIV-1 p24 gag Ag production with a commercially available kit (Abbott labs, Pomezia, Italy) as described before. The p24 gag Ag evaluation was repeated at later time points in selected experiments; the geometric mean of p24 gag Ag production of replicates in each experiment was used to determine the effective drug concentration where 50% and 90% of viral replication is inhibited (EC_50 _and EC_90_, respectively), by linear regression of the log of the percent HIV-1-p24 production (compared to untreated controls) versus the log of the drug concentration.

#### Assessment of antiviral drug activity in chronically infected M/M

M/M were defined chronically infected when no new rounds of infection occurr in *in vitro *cultures and the p24 production remains stable. Our previous experience demonstrated that such status of chronical infection occurs starting from day 10 after virus challenge. For this purpose, M/M were challenged with 300 TCID_50_/ml of HIV-1_BaL _(in the absence of any drug) at day 0, and p24 gag Ag analysis was carried out from day 6 up to the point when at least two consecutive determinations showed stable production (around day 10–14 in all experiments performed for this purpose). At the time of chronical infection (hereinafter called day 0 for these experiments with chronically-infected M/M), M/M were carefully washed at least twice to remove any virus present in the supernatants, replenished with fresh complete medium containing various concentrations of MnTBAP (0.24–30 μM), Amprenavir (4 μM) or AZT (10 μM), and cultured under the same conditions as described before. Each drug concentration was run in triplicate or quadruplicate while positive controls were run in sextuplicate. Therefore, unless differently stated, drugs were then added at the time of chronical infection (i.e. day 10), and replaced each time of medium change (i. e. every 2 days).

#### Assessment of drug activity in acutely-infected PBL

PBL were plated in 48-well plates in the presence or absence of various concentrations of drugs, and challenged 30 minutes later with 300 TCID_50_/ml of HIV-1_IIIB_. After 2 hours cells were washed, counted, and plated with complete medium containing the appropriate drugs concentrations. Assessment of virus replication was performed by HIV-1-p24 ELISA.

#### Virus infectivity

Infectivity of virus particles produced by HIV-1-infected M/M was evaluated on M/M obtained from a different seronegative donor exposed to serial dilution of supernatants from drug treated or not-treated HIV-1-infected M/M. The TCID_50_/ml was calculated according to Reed and Muench method.

#### Western blot analysis

After cells were washed with phosphate-buffered saline (PBS, BioWhittaker, Walkersville, MD), they were lysed with 0.75% Triton X-100 lysis buffer containing 300 mM NaCl, 50 mM Tris-HCl, pH 7.4, 2 μL/mL DMSO, and a cocktail of protease inhibitors containing 10 μg/mL Leupeptin, 20 μg/mL Aprotinin, 25 μM *p*-nitrophenyl guanidinobenzoate (pNGb). After a ten minute incubation in lysis buffer at 4°C, the cell lysate was clarified by centrifugation for ten minutes at 10,000 rpm. Total protein concentration was determined using the BCA Assay (Pierce, Rockford, IL). Cell lysates were resuspended in SDS sample buffer containing 50 mM dithiotreitol (DTT). Cell lysates (2 μg) were then loaded in a 10% Bis-Tris polyacrylamide gel (Novex, San Diego, CA), after separation by SDS/PAGE, proteins were transferred electrophoretically to nitrocellulose membranes and detected with a monoclonal mouse antibody to HIV-1-p24 (Intracel, Cambridge, MA).

#### Immunocytochemical Staining

Immunocytochemical staining for nitrotyrosine was performed on treated or not treated M/M. M/M were fixed with 4% paraformaldeyde dissolved in 0.1% phosphate buffer (pH 7.4). Nonspecific staining was blocked with 3% normal goat serum in 0.5 M Tris-HCl, pH 7.4 containing 0.2% Tween 20 for 1 h at room temperature. All subsequent incubations were carried out in this buffer. For detection of nitrotyrosine immunoreactivity, cells were incubated overnight at 4°C with an anti-nitrotyrosine monoclonal Ab (Cayman, 1:500). Treatment with secondary antibody, A/B complex, and DAB were performed by the manufacturer's instructions (Vector ABC Elite Kit, Vector Laboratories).

#### Malondialdehyde Determinations

Malondialdehyde (MDA), used as a biochemical marker for lipid peroxidation, was measured by a method previously described [45]. Briefly, cells were homogenized in potassium chloride (1.15%) and frozen in liquid nitrogen. Chloroform (2 ml) was then added to each homogenate and then spun for 30 min. The organic layer of the sample was removed and dried under nitrogen gas and reconstituted with 100 μl of saline. MDA generation was evaluated by the assay of thiobarbituric acid (TBA)-reacting compounds. The addition of a solution of 20 μl of sodium dodecyl sulphate (SDS; 8.1%), 150 μl of 20% acetic acid solution (pH3.5), 150 μl of 0.8% TBA and 400 μl of distilled water, produced a chromogenic product which was extracted in n-butanol and pyridine. Then, the organic layer was removed and MDA levels read at 532 nm and expressed as nmol MDA/g prot.

#### Ultrastructural studies

Cells for electron microscopy were fixed in 2.5% glutaraldehyde in PBS pH7.4 at 4°C and then washed for 2 hours in PBS and post fixed in osmium tetroxide 1.33% for 2 hours at 4°C. After several washes in PBS, the cells were dehydrated in graded alcohol, transferred into toluene, and embedded in Epon 812 resin. The resin was allowed to polymerize in a dry oven at 60°C for 24 hours. Thin sections were cut with a glass knife Reichert microtome, stained with toluidine blue and examined on Axioscope microscope. Ultra-thin sections were cut on a Reichert microtome using a diamond knife, stained with uranyl-acetate-lead-hydroxide and evaluated and photographed on a Philips electron microscope CM 10 (Philips).

#### Statistics

The differences in the EC_50 _in different cell populations and under different conditions of infection were assessed using the Student's t test. Results are given as mean ± sem. Statistical analysis was performed using ANOVA followed by Student-Newman-Keuls. P < 0.05 was considered statistically significant.

## Competing interests

The author(s) declare that they have no competing interests.

## Authors' contributions

SA conceived of the study, carried out the virus infectivity assays and drafted the manuscript. CM conceived of the study, carried out the immunocytochemistry and biochemical assays and drafted the manuscript. AR participated in the infectious assays and performed the statistical analysis. MP carried out the infectious studies, cell death analysis and helped to draft the manuscript. TG, LM and AM carried out the electron microscopy studies. CFP conceived of the study, and participated in its design and coordination. VM conceived of the study, and participated in its coordination and helped to draft the manuscript. All authors read and approved the final manuscript.
